# Ectopic Cushing’s Syndrome in Advanced Small-Cell Lung Cancer (SCLC): Clinical Challenges and Therapeutic Insights

**DOI:** 10.3390/cancers17101611

**Published:** 2025-05-09

**Authors:** Aleksandra Gamrat-Żmuda, Mari Minasyan, Piotr J. Wysocki, Alicja Hubalewska-Dydejczyk, Aleksandra Gilis-Januszewska

**Affiliations:** 1 Department of Endocrinology, Jagiellonian University Medical College, 30-688 Kraków, Poland; aleksandra.gamrat@gmail.com (A.G.-Ż.); mari.minasyjan@gmail.com (M.M.); alahub@cm-uj.krakow.pl (A.H.-D.); 2 Doctoral School of Medical and Health Sciences, Jagiellonian University Medical College, 31-530 Kraków, Poland; 3 Department of Oncology, University Hospital, 31-501 Kraków, Poland; piotr.wysocki@uj.edu.pl

**Keywords:** ectopic Cushing’s syndrome (ECS), Small-cell lung cancer (SCLC), hypercortisolemia, hypokalemia

## Abstract

Background/Objectives: Ectopic Cushing’s syndrome (ECS) is a rare, life-threatening condition caused by uncontrolled ACTH secretion from tumors, most commonly small-cell lung cancer (SCLC). ECS is traditionally reported in 1–6% of SCLC cases; however, recent data suggest it may be much higher. This study compares the clinical presentation of SCLC-related ECS (ECS-SCLC) with other ECS etiologies and analyzes the diagnosis, treatment, and outcomes of ECS-SCLC. Methods: We retrospectively analyzed the records of 39 ECS patients diagnosed between 2000 and 2024 at a tertiary endocrinology center. Seven cases (18%) were due to SCLC. Diagnosis was based on clinical signs, biochemical testing, imaging, and histopathology. Results: ECS-SCLC patients (five men, two women; median age 61), compared to other ECS etiologies, had a shorter time to diagnosis (median 1 vs. 2 months; *p* = 0.03), worse general condition (ECOG 4 vs. 3; *p* = 0.01), greater muscle weakness (Lovett scale median 2[IQR 1–2] vs. 2[IQR 2–3]; *p* = 0.04), more severe hypokalemia (2.12 vs. 2.7 mmol/L; *p* = 0.03), and required higher potassium supplementation (200 vs. 120 mEq/day; *p* = 0.001). All ECS-SCLC patients experienced weight loss (median 5 kg). Cortisol-lowering therapy (metyrapone or osilodrostat) was initiated in six patients (mean initiation time 3.7 days), leading to clinical improvement. Oncological treatment (chemotherapy or radiotherapy) was administered in five patients after stabilization. The median follow-up time was 3 months. Conclusions: Early recognition of ECS-SCLC and a multidisciplinary approach are critical. Severe hypokalemia and muscle weakness should prompt timely evaluation for hypercortisolism. Cortisol-lowering therapy may improve clinical status and facilitate oncological treatment.

## Simple Summary

Ectopic Cushing’s syndrome (ECS) is a rare and serious condition caused by excessive production of adrenocorticotropic hormone (ACTH) by tumors, leading to dangerously high cortisol levels. Small-cell lung cancer (SCLC) is one of the most common causes of ECS, but its incidence may be underestimated. Patients with SCLC-related ECS often present with nonspecific but severe symptoms such as muscle weakness, weight loss, and life-threatening hypokalemia, which can delay diagnosis. In this study, we analyze symptoms, diagnostic challenges, and treatment effectiveness in SCLC-ECS. Managing cortisol levels can improve patients’ conditions and even enable oncological treatment. Our findings highlight the importance of early recognition of ECS in SCLC patients and the need for a multidisciplinary approach. Increasing awareness among oncologists and primary care physicians may lead to earlier diagnosis and better patient outcomes.

## 1. Introduction

Ectopic Cushing’s syndrome (ECS), also known as paraneoplastic Cushing’s syndrome, is a rare clinical condition associated with unregulated expression and secretion of adrenocorticotropic hormone (ACTH) by neuroendocrine tumors, irrespective of their location or aggressiveness. The most common causes of ECS are considered to be bronchial carcinoids and small-cell lung cancer (SCLC), followed by gastrointestinal neuroendocrine tumors, medullary thyroid cancer, and pheochromocytomas. Rare causes include malignant tumors such as breast, ovarian, and prostate cancer [1,2,3]. In 20% of cases, despite repeated imaging studies, the underlying cause of ECS remains unknown for many years [1,2]. Ectopic Cushing’s syndrome represents a significant clinical challenge requiring immediate diagnosis and treatment due to the risk of life-threatening complications resulting from severe hypercortisolemia [3,4]. The combination of ECS and tumor-induced immunosuppression can lead to serious infections and worsening of oncological outcomes [5,6]. Fewer than 140 cases of ECS caused by small-cell lung cancer (SCLC) have been documented to date [3], although SCLC is one of the most common causes of this clinical condition, accounting for 20–30% of all ECS cases [7,8]. However, the incidence of ECS in SCLC patients appears to be underestimated, with previous estimates indicating that ECS occurs in 1–6% of SCLC patients [9,10]. Most epidemiological data come from tertiary referral centers, introducing a potential bias in patient recruitment. Additionally, not all cases are likely to be recorded. Recent reports suggest that ECS in SCLC patients is often overlooked and may, in fact, affect up to 20–30% of advanced SCLC patients [6,11,12]. We aim to compare the clinical presentation of SCLC-related ECS with other ECS etiologies and analyze the diagnosis, treatment, and outcomes of ECS-SCLC. We hope that this article will help to optimize clinical practice by providing a comprehensive overview of this rare but significant syndrome.

## 2. Materials and Methods

We have retrospectively analyzed medical records of consecutive patients with ECS diagnosed and treated at a tertiary endocrinological referral center in southern Poland between 2000 and 2024. A total of 39 cases were identified. The diagnosis of ECS was based on clinical presentation, biochemical tests, and imaging studies. In some cases, additional diagnostic tests were performed, including corticotropin-releasing hormone (CRH)/desmopressin tests, the high-dose dexamethasone suppression test (HDDST), and bilateral inferior petrosal sinus sampling (BIPSS). In select cases, immunohistochemical staining of ACTH was used in histopathological examinations of the lesion identified as the ectopic source. Among the 39 ECS cases, 7 patients (18%) presented with SCLC as the underlying cause of ECS, with the diagnosis being confirmed by histopathological examination.

First, we compared the clinical presentation of ECS-SCLC patients with that of other ECS cases in our cohort by analyzing variables including sex, age, general clinical status (assessed using the ECOG scale), symptoms of Cushing’s syndrome (including initial manifestations), comorbidities, time to diagnosis of Cushing’s syndrome, key basic laboratory parameters (including electrolyte levels), potassium supplementation details, survival time or follow-up duration, and cause of death. The assessment of hypercortisolemia symptoms at the time of diagnosis included weight changes (over the preceding year), redistribution of adipose tissue, striae, plethora, edema, hirsutism (using Ferriman–Gallwey scale), skin thinning, bruising tendency, proximal muscle weakness (evaluated using Lovett’s scale), susceptibility to infections (defined as infections requiring hospitalization at the time of diagnosis or within the six months prior), psychotic symptoms, depression, and anxiety. The presence of comorbidities was also assessed: resistant hypertension (defined according to the American Heart Association: failure to achieve target blood pressure values despite the use of at least three different antihypertensive drugs in optimal doses, including a diuretic), osteoporosis, and type 2 diabetes. Initial symptoms were defined as those first reported by the patient or first identified by medical personnel. Time to diagnosis was defined as the interval between the onset of initial symptoms and the diagnosis of endogenous hypercortisolemia. Survival time or follow-up duration was calculated from the date of diagnosis of endogenous hypercortisolemia.

In the next stage of our analysis, we conducted a detailed evaluation of the diagnostic and therapeutic course in patients with ECS-SCLC. For each case, we assessed laboratory test results (including cortisol, ACTH, and liver function parameters), imaging results, the applied therapeutic strategy (encompassing both adrenostatic and oncological treatment), and the response to the therapy. In order to objectively assess the change in clinical status, the time to improvement in muscle strength was calculated, defined as the time from initiation of treatment for hypercortisolism to improvement in muscle strength, as measured by the Lovett scale, defined as an increase of at least 1 degree. Moreover, the time to kalemia response was defined as the time from initiation of treatment for hypercortisolism to any reduction in potassium supplementation or achieving normokalemia with the previously used potassium supplementation.

Frequencies of clinical parameters in the group are expressed as percentages. In order to summarize individual numerical parameters in the patient group, the mean was defined. Statistical analysis was performed using IBM SPSS Statistics software (version 29). Considering the sample size and non-normal distribution of continuous variables, nonparametric methods were used. Comparisons of categorical variables were performed using the Fisher exact test, and for continuous variables, the Mann–Whitney U test was used. A *p*-value of <0.05 was considered statistically significant. The study was approved by the Ethics Committee of Jagiellonian University (approval number: 118.0043.1.75.2024). Given the retrospective nature of the study, patient consent was not required.

## 3. Results

### 3.1. Clinical Presentation

Among 39 patients with ECS (18 women, 21 men; mean age 60 years, range: 16–87 years), we identified small-cell lung cancer as the source of ACTH in 7 patients (18%). Other etiologies included neuroendocrine tumors of the gastrointestinal tract (9/39, 23%), carcinoid tumors of the lung or thymus (6/39, 15.4%), medullary thyroid cancer (3/39, 7.7%), pheochromocytoma (2/39, 5.1%), maxillary sinus papilloma (1/39, 2.6%), esthesioneneuroblastoma (1/39, 2.6%), other malignant tumors (7/39, 18%), and in 3 cases, the source of ECS remained occult (Table 1).

The ECS-SCLC subgroup included five men and two women, with a median age of 66 years (IQR 62–68), which was not significantly different from the remaining cohort in terms of age or sex distribution. The time to diagnosis in ECS-SCLC ranged from 0.5 to 3 months and was statistically significantly lower than in patients with other causes of ECS (median: 1 month vs. 2 months, *p* = 0.03). A summary of the comparative analysis of the clinical presentation between patients with ECS caused by SCLC and those with ECS of other etiologies is presented in Table 2. At the time of diagnosis, patients with ECS-SCLC were in significantly worse overall condition, as assessed by the ECOG performance status, compared to patients with EAS of other etiologies (median 4 vs. 3, *p* = 0.01). The initial symptoms of ectopic Cushing’s syndrome leading to diagnosis and urgent hospitalization in patients with ECS-SCLC were profound muscle weakness (<3 on the Lovett scale), edema, severe treatment-resistant hypokalemia, and weight loss. Hypokalemia as an initial symptom occurred significantly more frequently in the ECS-SCLC group compared to patients with ECS of other etiologies (100% vs. 44%, *p* = 0.01). Although hypokalemia was severe across all patients with ECS, serum potassium levels were significantly lower in the ECS-SCLC group compared to those with other etiologies (2.12 mmol/L vs. 2.7 mmol/L, *p* = 0.03), and these patients required higher potassium supplementation (200 mEq/day vs. 120 mEq/day, *p* = 0.001). Muscle weakness expressed by the Lovett scale was also more severe in patients with ECS-SCLC than in patients with ECS of other etiology (median 2, IQR 1–2 vs. median 2 IQR 2–3; *p* = 0.04. Common symptoms observed in patients with SCLC-related ECS also included weight loss (100%, median 5 kg), oedema (71%), easy bruising (57%), and increased tendency to infections (71%); however, their prevalence did not differ from that seen in other ECS etiologies (Table 2).

Bacterial infections (upper and lower respiratory tract infection, urinary tract infection, joint infection) occurred in five patients (1, 2, 4, 5, and 7) within one month before or during hospitalization, and two patients (5 and 7) developed sepsis (Table 3). There were no statistically significant differences between the ECS-SCLC group and the remaining cohort in the frequency of typical hypercortisolism-related features, including fat redistribution, striae, facial plethora, or the frequency of comorbidities associated with Cushing’s syndrome (Table 2). Classic features of Cushing’s syndrome among patients with ECS-SCLC were infrequent: redistribution of fat tissue was noted in three patients, plethora in three, and striae in one. Hypertension was present in all ESC-SCLC cases, while in four patients, this was classified as resistant hypertension and required multidrug therapy (3–5 antihypertensive drugs). Carbohydrate metabolism disorders were observed in five patients: one prediabetic patient, one diabetic patient treated with oral antidiabetic drugs, and three diabetic patients requiring intensive insulin therapy with high daily doses of insulin > 40 units (Table 3).

### 3.2. Diagnosis of ECS-SCLC

Hormonal evaluation confirmed significant hypercortisolemia in all patients with ECS caused by SCLC (serum cortisol: 22.7–192 μg/dL, mean 91.7 μg/dL; salivary cortisol: 0.3–24 μg/dL in three tested patients). Levels of ACTH ranged from 60 to 1237 pg/mL (mean 345.9 pg/mL), with one patient exceeding 20 times the upper normal limit (Table 3). The 1 mg dexamethasone suppression test was performed in five patients, all showing inadequate cortisol suppression (mean post-test cortisol: 59.5 μg/dL, mean relative decrease: 22.4%). Severe hypercortisolemia led to psychiatric symptoms in four patients (psychosis in two, depression in three). Diagnostic imaging was promptly conducted to identify the ectopic ACTH source, with confirmation of the diagnosis within 2 weeks to 2.5 months. Initial imaging findings varied: in patient 1-MRI of the pituitary (no abnormalities), in patient 2-abdominal CT (revealed metastatic lesions), and in patient 6-chest X-ray (unremarkable, later CT-confirmed primary lung lesion). In the remaining patients, whole-body CT successfully identified primary lesions. Small-cell lung cancer was confirmed cytologically or histopathologically via bronchoscopy, endoscopic ultrasound with biopsy, or pleural fluid analysis. All patients had metastatic disease, most commonly in the mediastinal lymph nodes (all patients) and adrenal glands (three patients) (Table 3).

### 3.3. Treatment Strategy and Results Among Patients with ESC-SCLC

A multidisciplinary team, including an endocrinologist, radiologist, oncologist, radiotherapy specialist, and psychologist, assessed all ECS-SCLC patients. Treatment for hypercortisolemia was initiated in six of these patients (diagnosed between 2022 and 2024). Patient 1 was diagnosed in 2009 but had not received any treatment for hypercortisolemia, according to the medical history, due to elevated liver function tests (ALT 172, AST 136 U/I). The mean time between diagnosis of ACTH-dependent Cushing’s syndrome and initiation of treatment for hypercortisolism was 3.7 days. Metyrapone was the first-line therapy for four patients, while osilodrostat was initiated in two patients. All of these patients presented with elevated liver function tests (LFTs) at the time of adrenostatic treatment initiation (greatest values were seen in patient 6: GGTP 21x upper limit of normal (ULN), ALT 6.2× ULN, AST 2.9× ULN). Among patients treated with metyrapone, patients 3-6 had a decrease in ALT, patient 5 had a decrease in AST, while patients 4 and 6 had an increase in AST. Among patients treated with osilodrostat, patients 3 and 7 had a decrease in LFTs, patients 2 and 4 had an increase in LFTs, and patient 5 had an intermittent increase in LFTs (Supplementary Materials).

**Figure 1:**
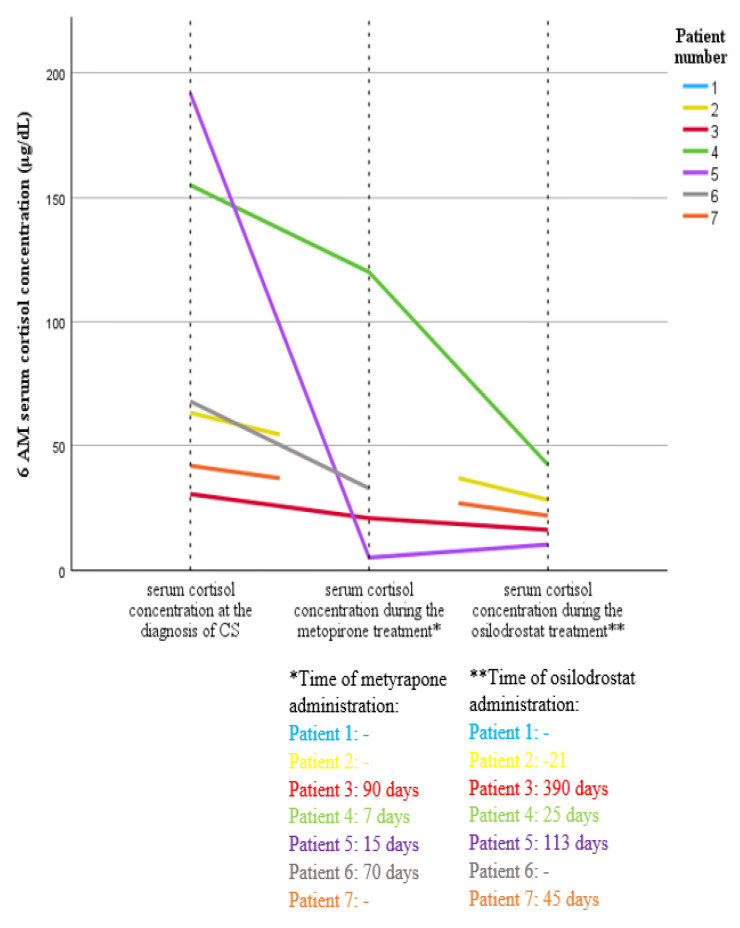
Variability in morning serum cortisol concentration at the time of diagnosis and during treatment with metyrapone and osilodrostat in patients with ECS in the course of SCLC.

**Figure 2:**
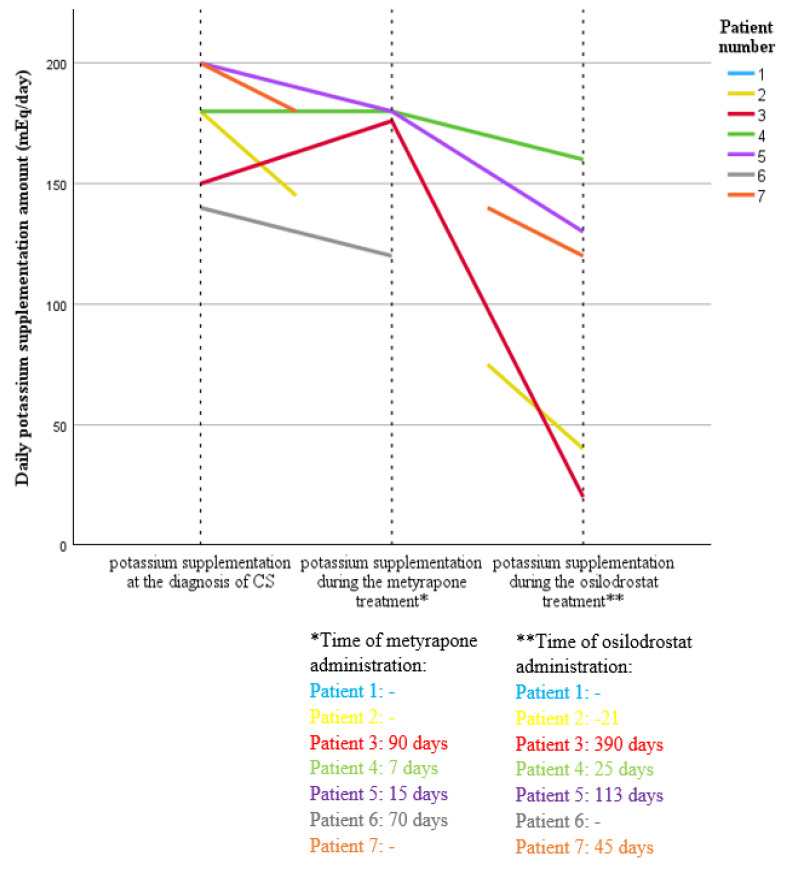
Variability in the need for potassium supplementation at the time of diagnosis and during treatment with metyrapone and osilodrostat in patients with ECS in the course of SCLC.

Patient 2 received osilodrostat for 21 days (max dose 4 mg/day), achieving a 55% reduction in cortisol levels (Figure 1) and a three-fold decrease in potassium supplementation (from 120 mEq/d to 40 mEq/d) (Figure 2).

Patient 7 was treated with osilodrostat (40 mg/day) in a “block and replace” regimen with hydrocortisone, leading to cortisol normalization (Figure 1) and a 40% reduction in potassium supplementation (Figure 2). Patient 6, treated exclusively with metyrapone for 70 days, showed a transient clinical improvement and a 52% reduction in cortisol levels (Figure 1); however, potassium supplementation was kept at the same dosage (140 mEq/d) (Figure 2). In three patients (patients 3, 4, and 5), sequential therapy was used, starting with metyrapone (average time of use 37.7 days) followed by osilodrostat (average time of use 176 days). In three patients, therapy was modified because of failure to achieve eucortisolism or insufficient clinical response (patients 3 and 4), while in one patient, treatment was modified due to poor treatment tolerance (patient 5) (Table 4).

In Patient 3, osilodrostat led to a 74% reduction in cortisol levels (Figure 1), decreased potassium supplementation (Figure 2), and a reduced number of antihypertensive drugs used (from 5 to 3). In patient 4, osilodrostat therapy following metyrapone treatment resulted in a 73% reduction in cortisol levels (Figure 1) and a minor decrease in potassium supplementation (from 180 mEq/d to 160 mEq/d) (Figure 2). The patient passed away 25 days after treatment initiation due to advanced primary disease. Patient 5 was switched to osilodrostat therapy (up to 10 mg/day) after 15 days on metyrapone, achieving nearly a 90% reduction in cortisol levels (Figure 1), reduced potassium supplementation (from 200 mEq/d to 120 mEq/d) (Figure 2), and decreased number of antihypertensive medications used (one less). Despite experiencing sepsis, osilodrostat therapy was maintained with hydrocortisone supplementation in a “block and replace” regimen. Metyrapone treatment (patients 3–6) led to an average cortisol reduction of 44%, with modest clinical improvement (mean potassium supplementation reduction: 12.2%). Osilodrostat treatment (patients 2–5, 7) resulted in a mean reduction in cortisol levels of 63.4% and a more than 50% decrease in potassium supplementation (mean reduction: 50.1%) (Figure 1 and Figure 2).

At the time of ECS diagnosis, all ECS-SCLC patients had an ECOG score of 4. The mean time to improvement in kalemia control, defined as the time from initiation of treatment for hypercortisolism to any reduction in potassium supplementation or achieving normokalemia with the previously used potassium supplementation, was 13.8 days. The mean time to improvement in muscle strength, defined as an increase of at least 1 point on the Lovett scale from the moment of treatment initiation for hypercortisolism, was 12.2 days (improvement in muscle strength was achieved in five patients). In four patients, there was an improvement in the ECOG score (from 4 to 3). Given the advanced stage of SCLC, all patients were eligible for palliative treatment (Table 4). Three patients qualified for palliative radiotherapy (mediastinal RTH in patients 2 and 6, brain RTH in patient 4), while two patients underwent chemotherapy (cisplatin+etoposide in patient 5 and carboplatin+etoposide+atezolizumab in patient 3). The mean time to implement oncological treatment was 24.4 days from the time of ECS diagnosis. Two patients were deemed unsuitable for oncological treatment and received best supportive care. Patient 3 showed a partial tumor response and later discontinued osilodrostat after 13 months due to complete ECS remission and long-term SCLC control, continuing atezolizumab maintenance therapy without ECS recurrence for 31 months. All remaining patients died mainly due to progression of primary disease, with a mean survival time of 2.3 months. All of these patients had been receiving treatment for hypercortisolism until death. Details regarding response to oncological treatment and causes of death are presented in Table 4.

## 4. Discussion

All of the patients with ECS in our analysis were diagnosed at a tertiary referral endocrinology center. ECS caused by SCLC represented 18% of all 39 ECS cases diagnosed between 2000 and 2024 at this unit. This percentage is lower than the 20–30% prevalence of ECS caused by SCLC reported in the literature [7,8], supporting the notion that ECS in SCLC patients may be overlooked and that such patients are infrequently referred to endocrinology departments [10,11]. Small-cell lung cancer patients diagnosed with ECS induced by ectopic ACTH production represent a distinct subgroup within the Cushing’s syndrome spectrum and among other causes of ectopic ACTH secretion. In our cohort, patients with ECS due to SCLC demonstrated a significantly more severe clinical profile compared to those with other ECS etiologies, characterized by poorer performance status on the ECOG scale, more pronounced muscle weakness, and more severe hypokalemia, necessitating consequently higher doses of potassium supplementation. Unlike typical presentations of Cushing’s syndrome—characterized by features such as striae, weight gain, and fat tissue redistribution—patients with SCLC-related ECS rarely exhibited these symptoms. Instead, their clinical profile was dominated by catabolic manifestations, including significant weight loss and oedema. The presence of catabolic symptoms, which are considered atypical for Cushing’s syndrome, has been well documented in SCLC-related ECS [3,11,12]. Li et al. identified hypokalemia as the most common symptom, occurring in nearly 97% of cases of SCLC-ECS [3], followed by hypertension (69%) and carbohydrate metabolism disorders (61%). Similar to our study, the most frequently observed physical symptoms were muscle weakness (67%) and edema (59%).

In all presented ECS-SCLC patients, the severity of the general condition, as well as the intensity and rapid progression of symptoms, necessitated hospitalization. As a result, the diagnosis of Cushing’s syndrome was established more promptly compared to other ECS cases. Severe hypokalemia and marked muscle weakness, observed in all ECS-SCLC patients, proved to be key diagnostic indicators that prompted timely hospitalization and endocrinological assessment. Castro et al. reported that the symptoms of ectopic Cushing’s syndrome are a continuum, but the hypercortisolemia in SCLC patients is often very rapid and aggressive [7].

After confirming severe ACTH-dependent hypercortisolemia with rapidly progressing symptoms, imaging studies were then conducted to identify the source of ectopic ACTH secretion, making differential laboratory diagnostics unnecessary. This approach is a standard practice used when the suspected ectopic source is a malignant neoplasm, as these cases typically, though not always, present with pronounced symptom severity and advanced hypercortisolemia [7,13,14].

All patients in our analysis were diagnosed with advanced (disseminated) SCLC, suggesting that the occurrence of clinically evident SCLC-ECS may be a late stage in the disease development. Most likely, earlier symptoms were subtle and remained unnoticed by the patients or misdiagnosed by primary care physicians [5,6]. Severe hypokalemia can be a trigger for other symptoms, such as muscle weakness, which is often observed in patients with ECS, and could be the most important trigger of life-threatening conditions in patients with ECS [15]. Low potassium levels cause dangerous cardiac arrhythmias (e.g., ventricular tachycardia of the torsade de pointes type), neurological disorders such as hyperactivity or apathy, and hypokalemia-induced muscle weakness, which in extremely low potassium levels can progress to paralysis leading to paralytic ileus. Additionally, it can precipitate rhabdomyolysis, which manifests as muscle tenderness and swelling [16,17,18]. Awareness of this unique clinical presentation of CS is essential for the accurate diagnosis of ECS in the context of SCLC. Educating primary care physicians, internists, and oncologists about the alarm symptoms of hypercortisolemia—such as hypokalemia, hypertension, and carbohydrate metabolism disorders—in patients suspected of having lung cancer, along with the awareness that typical Cushing’s syndrome symptoms may be absent in SCLC-ECS, may lead to earlier diagnosis and treatment of ECS. A multidisciplinary approach that includes referral to an endocrinologist should be the standard of care for SCLC patients presenting with ECS symptoms.

In each of our patients, the treatment of hypercortisolemia was effective. Therapy led to a decrease in morning cortisol levels, better control of potassium levels, the possibility of discontinuing some antihypertensive medications, and, in most cases, an overall improvement in the patient’s clinical condition, seen as improvements in muscle strength and ECOG score. In some of these cases, the improvements allowed the implementation of oncological treatment. Moreover, it is highly plausible that this treatment improved the outcomes of SCLC patients, aligning with the positive outcomes of osilodrostat treatment in ECS patients documented in a prospective study by Dormoy et al. [8]. The case of patient no. 3, which was recently published [9], marks the first reported instance of complete remission of hypercortisolemia in a patient with severe SCLC-ECS. Early intervention, leading to effective hypercortisolemia control, improved the patient’s performance status and facilitated the use of chemotherapy. This combined approach significantly slowed disease progression and resulted in long-term survival of more than two years (as of this publication).

Previous reports have shown that there is a tendency to prolong survival when a high cortisol level is controlled before initiating treatment [19,20,21]. In addition, Li et al. [3] noted that immune checkpoint inhibitors (ICIs), such as atezolizumab, which was used in patient 3, can significantly improve overall survival. Therefore, it is essential to achieve a general condition in which immunochemotherapy can be applied. Importantly, there are isolated reports that immunochemotherapy may induce CS, which should also be considered when treating patients with SCLC, as immunochemotherapy is presently a common, first-line treatment [22]. A reduction in the amount of necessary potassium supplementation appears to be a good marker of the effectiveness of the treatment of hypercortisolemia in ECS. The response in the form of potassium level control reduces the risk of life-threatening complications of hypokalemia caused by hypercortisolemia [23,24].

It is worth noting that elevated liver enzymes are a common baseline finding in CS patients and are not a contraindication for metyrapone or osilodrostat implementation [25,26,27]. The lowering of cortisol levels may lead to liver function improvement. Most of the presented cases demonstrated LFT amelioration upon therapy for hypercortisolemia. Cases where we observed an increase in LFTs were mostly connected with infections and general deterioration due to neoplastic disease progression. Situations with separate AST elevation could have been associated with an extrahepatic origin of the enzyme (e.g., myopathy).

Our study is limited by its retrospective design, which may introduce selection bias and limit the generalizability of the findings. Another limitation is the small number of patients analyzed. However, these patients represent all cases diagnosed and treated over 25 years at a tertiary referral center in the endocrinology department, highlighting the rarity of this disease. This underscores the need for multicenter studies to draw more reliable conclusions. Another limitation, which affects the objective assessment of treatment for hypercortisolism in SCLC-related ECS, was the highly variable duration of cortisol synthesis-blocking drug use among patients. This variability was influenced by the severity of the condition and the patient’s survival period. Prospective comparative studies are needed to evaluate the effectiveness of different cortisol synthesis inhibitors in SCLC-related ECS, as well as their interactions with oncological treatment.

## 5. Conclusions

In summary, early diagnosis and effective multidisciplinary treatment of SCLC-related ECS, particularly rapid treatment with cortisol synthesis blockers, appears to be essential for improving patient condition, reducing life-threatening complications, and facilitating the implementation of systemic oncological treatment.

## Figures and Tables

**Table 1:** A summary of ECS etiology in patient cohort.

ECS Etiology	N = 39 (Percentage)
SCLC	7 (18%)
GEPNEN	9 (23%)
-Pancreatic NET	7
-Gastric NET	1
-Intestinal NET	1
Carcinoid	6 (15.4%)
-Lung carcinoid	5
-Thymic carcinoid	1
Medullary thyroid cancer	3 (7.7%)
Pheochromocytoma	2 (5.1%)
Other malignant tumors	7 (18%)
-Ovarian cancer	2
-Gastric adenocarcinoma	1
-Lung adenocarcionoma	1
-Uterine clear cell carcinoma	1
-Pancreatic tumor ^1^	1
-Bladder tumor ^1^	1
Other tumors	2 (5.1%)
-Maxillary sinus papilloma	1
-Esthesioneneuroblastoma	1
Occult source ^2^	3 (7.7%)

^1^ Tumor identified in imaging results, but no histopathological examination available. ^2^ Ectopic Cushing’s syndrome confirmed by biochemical tests, ACTH source not identified by repetitive imaging evaluation. Abbreviations: ECS, ectopic Cushing’s syndrome; SCLC, small-cell lung cancer; GEPNEN, gastroenteropancreatic neuroendocrine neoplasm.

**Table 2:** A comparison of clinical features between patients with ECS caused by SCLC and those with other etiologies of ECS.

Variable ^1^	SCLC N = 7	Other ECS N = 32	*p*-Value ^2^
Age (years)	66 [62–68]	61 [45.5–70.8]	0.26
Women	2 (29%)	16 (50%)	0.42
**Time to diagnosis (months)**	**1 [1–2]**	**2 [1.13–4]**	**0.03**
Death	6 (86%)	20 (63%)	0.39
Time of observation (months)	3 [2–4]	12 [2–47.8]	0.13
**ECOG scale ^3^ at the diagnosis**	**4 [4–4]**	**3 [1–4]**	**0.01**
BMI	27 [22.6–36]	26.4 [22.2–34.9]	0.67
**Cushing’s syndrome symptoms**			
Weight gain	0 (0%)	11 (34%)	0.16
Weight loss	7 (100%)	19 (59%)	0.07
Weight change (kg)	−5 [(−8)–(−5)]	−4 [−7.8–9.3]	0.16
Fat tissue redistribution	4 (57%)	19 (59%)	0.62
Striae	1 (14%)	7 (22%)	1.00
Oedema	5 (71%)	22 (69%)	1.00
Plethora	3 (43%)	18 (56%)	0.64
**Lovett scale**	**2 [1–2]**	**2 [2–3]**	**0.04**
Tendency to bruise	4 (57%)	22 (69%)	0.67
Tendency to infection	5 (71%)	21 (66%)	1.00
**Electrolyte disturbances**			
Na+ concentration (mmol/L)	**144 [138–148]**	**144.5 [142–148]**	0.48
**K+ concentration (mmol/L)**	**2.12 [1.9–3.4]**	**2.7 [2.3–3.5]**	**0.03**
**Kalium supplementation (mEq/day)**	**200 [150–200]**	**120 [65–160]**	**0.001**
**Comorbidities**			
Hypertension	7 (100%)	29 (91%)	1.00
Diabetes mellitus	4 (57%)	22 (69%)	0.67
Hyperlipidemia	3 (43%)	18 (56%)	0.38
**Initial symptoms ^4^**			
**Hypokalemia**	**7 (100%)**	**13 (44%)**	**0.01**
Oedema	4 (57%)	9 (28%)	0.19
Muscle weakness	3 (43%)	12 (38%)	1.00
Weight loss	1 (14%)	5 (16%)	1.00

^1^ Continuous variables are present as median and interquartile range (IQR), and categorical variables as frequency (n) and percentage (%). ^2^ Normality of data distribution was evaluated using the Shapiro–Wilk test. Categorical variables were compared using Fisher’s exact test, while continuous variables were analyzed with the Mann–Whitney U test. ^3^ ECOG—The Eastern Cooperative Oncology Group Performance Status Scale; 0—fully active, able to carry on all pre-disease activities without restriction; 1—restricted in physically strenuous activity but ambulatory and able to perform light work; 2—ambulatory and capable of self-care but unable to carry out any work activities; up and about more than 50% of waking hours; 3—capable of only limited self-care; confined to bed or chair more than 50% of waking hours; 4—completely disabled; cannot carry out any self-care; totally confined to bed or chair; 5—deceased. ^4^ Defined as symptoms first reported by the patient or first identified by medical personnel. The bold was used to highlight statistically significant differences (*p* < 0.05).

**Table 3:** A summary of diagnostic data of ECS patients in the course of SCLC.

Patient Number	Gender (F-Female, M-Male)	Age at the Diagnosis (Years)	Initial Symptoms ^1^ 1. Hypokalemia 2. Muscle Weakness 3. Oedema 4. Reistant Hypertension 5. Weight Loss	Weight Changes Before Diagnosis (kg; “-” Means Weight Loss)	Other Symptoms 2. Muscle Weakness 4. Resistant Hypertension 5. Weight Loss 6. Fat Tissue Redistribution 7. Plethora 8. Striae 9. Skin Thinning 10. Tendency to Bruise 11. Tendency to Infection 12. Psychotic Symptoms 13. Depression	Time to Diagnosis of Hypercortisolemia ^2^ (Months)	Histopathological Diagnosis (Material)	TNM Scale	Size of Primary Lesion (mm)	Site of Metastases 1. Lymph Nodes 2. Liver 3. Bones 4. Adrenal Glands 5. Central Nervous System 6. Subcutaneous Tissue	6 AM Cortisol Concentration at the Diagnosis (ug/dL)	ACTH Concentration at the Diagnosis (pg/mL; N: 5–56)	Potassium Concentration at the Diagnosis (mmol/L)
1.	M	55	1. Hypokalemia 2. Muscle weakness	−8	5. Weight loss 11. Tendency to infection 12. Psychotic symptoms	1	SCLC (sample taken during EUS)	IVB-T4N3M1c	80 × 100 × 120	1. thoracic lymph nodes 4. adrenal glands	84	394	1.9
2.	M	74	1. Hypokalemia 4. Resistant hypertension	no information	2. Muscle weakness 5. Weight loss 11. tendency to infection	3	SCLC (sample taken during bronchoscopy)	IVB T4N3M1c	91 × 56 × 72	1. thoracic lymph nodes 4. adrenal glands	63.4	113.7	1.4
3.	M	65	1. Hypokalemia 3. Oedema	−8	2. Muscle weakness 4. Resistant hypertension 5. Weight loss 6. Fat tissue redistribution 7. Plethora	1	SCLC (sample taken during bronchoscopy)	IIIC-T3N3M0	52 × 26 mm	1. Thoracic lymph nodes	37.4	222	2.16
4.	M	66	1. Hypokalemia 3. Oedema 5. Weight loss	−11	2. Muscle weakness 4. Resistant hypertension 5. Weight loss 6. Fat tissue redistribution 7. Plethora 9. Skin thinning 10. Tendency to bruise 11. Tendency to infection 13. Depression	0.5	SCLC (sample taken during bronchoscopy)	IVB-T1cN3M1c	26 × 12	1. thoracic lymph nodes 3. bones 5. CNS	155	60	2.38
5.	F	62	1. Hypokalemia 2. Muscle weakness 3. Oedema	−5	5. Weight loss 6. Fat tissue redistribution 9. Skin thinning 10. Tendency to bruise 11. Tendency to infection 12. Psychotic symptoms	1	SCLC (sample taken during bronchoscopy)	IVB-T1cN3M1c	28 × 25	1. thoracic lymph nodes 2. liver 3. bones 4. adrenal glands	192	1237	1.9
6.	M	68	1. Hypokalemia 3. Oedema	−4	2. Muscle weakness 4. Resistant hypertension 5. Weight loss 9. Skin thinning 10. Tendency to bruise 11. Psychotic symptoms	2	SCLC (sample taken during bronchoscopy)	IVB-T4N3M1c	72 × 101 × 122	1. thoracic lymph nodes 2. liver 4. adrenal glands	68	223.6	2.34
7.	F	68	1. Hypokalemia 2. Muscle weakness	−5	3. Oedema 4. Resistant hypertension 5. Weight loss 6. Fat tissue redistribution 7. Plethora 8. Striae 9. Skin thinning 10. Tendency to bruise 11. Tendency to infection 13. Depression	1	SCLC [pleural fluid]	IVB- T?N3M1c	no information	1. mediastinal, paraaortic lymph nodes 2. liver 7. subcutaneous tissue of the abdomen	45.9	171	2.97

^1^ Symptoms that were diagnosed first or the patient reported first. ^2^ Time from appearance of first symptoms to the diagnosis of hypercortisolemia. Abbreviations: ECS, ectopic Cushing’s syndrome; ACTH, adrenocorticotropic hormone; SCLC, small-cell lung cancer; EUS, endoscopy ultrasonography.

**Table 4:** Summary of treatment data of patients with ECS in the course of SCLC.

Patient Number	ECOG ^1^ scale Assessment at ECS Diagnosis	Treatment of Hypercortisolemia (Maximal Dose Used)	Time from ECS Diagnosis to Hypercortisolemia Treatment Introduction (Days)	Total Duration of Treatment for HYPERCORTISOLISM (Days)	Time to Improve Muscle Strength ^2^	Time to Kalemia Response ^3^	Oncological Treatment	ECOG Scale Assessment Before Oncological Treatment	Time from ECS Diagnosis to Oncological Treatment Introduction	Total Duration of Oncological Treatment	RECIST 1.1 CR-Complete Response PR-Partial Response (% Reduction in Tumor Size) SD-Stable Disease PD-PROGRESSIVE disease (What Kind)	ECOG Scale Assessment During Oncological Treatment (Assessment Time ^4^)	Death (D), Survival Time/Follow-Up Time (Months)	Cause of Death
1.	4	-	-	-	-	-	-	-	-		-	-	D, 3	1. progression of the primary disease 2. infection
2.	4	1. Osilodrostat (4 mg)	4	1.21	10	5	RTH	4	20	2 days	no imaging tests during therapy	5 (2 days)	D, 1	1. progression of the disease 2. infection—sepsis
3.	4	1. Metyrapone (3250 mg) 2. Osilodrostat (5 mg)	6	1. 90 2. 390	18	21	CHT—carboplatin, etoposide, atezolizumab	3	49	31 months, 30 cycles, ongoing	PR (71%)	1 (4 months)	33, ongoing	-
4.	4	1. Metyrapone (1750 mg) 2. Osilodrostat (8 mg)	7	1. 7 2.25	15	29	Brain RTH-20 Gy in 5 fractions	3	21	8 days, death 2 weeks after RTH	no imaging tests during therapy	5 (2 weeks)	D, 1.5	1. progression of the primary disease
5.	4	1. Metyrapone (3000 mg) 2. Osilodrostat (10 mg)	1	1.15 2.113	11	17	CHT—cisplatine, etoposide	3	24	2.5 months, 4 cycles	PD (increased number and size of metastatic lesions in the liver)	2 (3 weeks); 5 (2.5 months)	D, 4	1. progression of the primary disease 2. infection—sepsis
6.	4	1. Metyrapone (1000 mg)	3	1.70	7	5	Mediastinal RTH	3	8	2.5 months	no imaging tests during therapy	3 (3 weeks); 5 (2.5 months)	D, 3	1. progression of the primary disease
7.	4	1. Osilodrostat (40 mg)	1	7.45	-	6	-	-	-	-	-	-	D, 1.5	-

^1^ ECOG—The Eastern Cooperative Oncology Group Performance Status Scale; 0—fully active, able to carry on all pre-disease activities without restriction; 1—restricted in physically strenuous activity but ambulatory and able to perform light work; 2—ambulatory and capable of self-care but unable to carry out any work activities; up and about more than 50% of waking hours; 3—capable of only limited self-care; confined to bed or chair more than 50% of waking hours; 4—completely disabled; cannot carry out any self-care; totally confined to bed or chair; 5—deceased. ^2^ Time from the initiation of treatment for hypercortisolism to improvement in muscle strength, as measured by the Lovett scale, defined as an increase of at least 1 degree. ^3^ Time from the initiation of treatment for hypercortisolism to any reduction in potassium supplementation or achieving normokalemia with the previously used potassium supplementation. ^4^ Time measured from the initiation of oncological treatment. Abbreviations: ECS, ectopic Cushing’s syndrome; SCLC, small-cell lung cancer; ECOG, Eastern Cooperative Oncology Group scale; CHT, chemotherapy; RTH, radiotherapy.

## Data Availability

Data supporting the findings of this study are available upon reasonable request from the corresponding author. Due to privacy and ethical restrictions, the datasets are not publicly accessible.

## Supplementary Materials

The following supporting information can be downloaded at: https://www.mdpi.com/article/10.3390/cancers17101611/s1, Supplementary Materials: The table shows changes of liver function tests during the treatment of hypercortisolemia with metyrapone and osilodrostat.

## Abbreviations

The following abbreviations are used in this manuscript:

ECS	Ectopic Cushing’s Syndrome
ACTH	Adrenocorticotropic Hormone
SCLC	Small-Cell Lung Cancer
GEPNEN	Gastroenteropancreatic neuroendocrine neoplasm
CRH	Corticotropin-Releasing Hormone
HDDST	High-Dose Dexamethasone Suppression Test
BIPSS	Bilateral Inferior Petrosal Sinus Sampling
LFT	Liver Function Tests
ULN	Upper Limit of Normal
MRI	Magnetic Resonance Imaging
CT	Computed Tomography
EUS	Endoscopic Ultrasound
UFC	Urine-Free Cortisol
ECOG	Eastern Cooperative Oncology Group Performance Status Scale
RTH	Radiotherapy
CHT	Chemotherapy
