# Supplementary material for: Ectopic Cushing’s Syndrome in Advanced Small-Cell Lung Cancer (SCLC): Clinical Challenges and Therapeutic Insights
Source: Cancers (Basel). 2025 May 9;17(10):1611. doi: 10.3390/cancers17101611 (PMC12109603; doi:10.3390/cancers17101611)
Supplement: Supplementary file 1 [file cancers-17-01611-s001.zip › cancers-3631055-supplementary.pdf]

Supplementary material. The table shows changes of liver function tests during the treatment of hypercortisolemia with metyrapone and osilodrostat.

Patient number	Liver enzymes concentration at the diagnosis<br>1.ALT U/I [N: 10 - 35]<br>2.AST U/I [N:10 - 35]<br>3.GGTP U/l [N:8 - 61]<br>4.Bilirubine µmol/l [N:0 - 21.00]	Liver enzymes concentration during treatment of hypercortisolemia with metyrapone. (assessed between 6th and 30th day of drug use; for Patient 3-6 results the results are for the moment of metyrapone stop and before osilodrostat start)<br>1.ALT U/I [N: 10 - 35]<br>2.AST U/I [N:10 - 35]<br>3.GGTP U/l [N:8 - 61]<br>4.Bilirubine µmol/l [N:0 - 21.00]	Total time of metyrapone administration (days)	Liver enzymes concentration during treatment of hypercortisolemia with osilodrostat (assessed between 6th and 21th day of drug use)<br>1.ALT U/I [N: 10 - 35]<br>2.AST U/I [N:10 - 35]<br>3.GGTP U/l [N:8 - 61]<br>4.Bilirubine µmol/l [N:0 - 21.00]	Total time of osilodrostat administration (days)
1.	1. 172<br>2. 136<br>3. 220<br>4. 15.9				
2.	1. 149<br>2. 68<br>3. 631<br>4. 22.7			1. 237<br>2. 185	21
3.	1. 118<br>2. 38<br>3. 92<br>4. 6.85	1. 25<br>2. 18<br>3. 64<br>4. 57		1. 18<br>2. 15<br>3. 55<br>4. 1.6	390
4.	1. 95<br>2. 62<br>3. 100<br>4. 13.6	1. 81<br>2. 91<br>3.61<br>4. 7.7	7	1. 99<br>2.177<br>3. 63<br>4. 9.3	25
5.	1. 217<br>2. 100<br>3. 472<br>4. 16.2	1. 29<br>2. 55<br>3. 695<br>4. 7.17	15	1. 126<br>2. 111<br>3. 742<br>4. 6.04	113
6.	1. 221<br>2. 123<br>3. 1303<br>4. 14.10	1.182<br>2. 161<br>3. -<br>4. -	70		
7.	1. 92<br>2. 39<br>3. -<br>4. 16.5			1. 33<br>2. 39<br>3. -<br>4. 10.10	32
